# Relevance of oxidative stress in inhibition of eIF2 alpha phosphorylation and stress granules formation during Usutu virus infection

**DOI:** 10.1371/journal.pntd.0009072

**Published:** 2021-01-25

**Authors:** Ana-Belén Blázquez, Miguel A. Martín-Acebes, Teresa Poderoso, Juan-Carlos Saiz

**Affiliations:** 1 Department of Biotechnology, Instituto Nacional de Investigación y Tecnología Agraria y Alimentaria (INIA), Madrid, Spain; 2 Molecular Virology Group, Department of Experimental and Health Sciences, Universitat Pompeu Fabra, Barcelona, Spain; National Research Centre, EGYPT

## Abstract

Usutu virus (USUV) is an African mosquito-borne flavivirus closely related to West Nile, Japanese encephalitis, Zika, and dengue viruses. USUV emerged in 1996 in Europe, where quickly spread across the continent causing a considerable number of bird deaths and varied neurological disorders in humans, including encephalitis, meningoencephalitis, or facial paralysis, thus warning about USUV as a potential health threat. USUV replication takes place on the endoplasmic reticulum (ER) of infected cells, inducing ER stress and resulting in the activation of stress-related cellular pathways collectively known as the integrated stress response (ISR). The alpha subunit of the eukaryotic initiation factor eIF2 (eIF2α), the core factor in this pathway, is phosphorylated by stress activated kinases: protein kinase R (PKR), PKR-like endoplasmic reticulum kinase (PERK), heme-regulated inhibitor kinase (HRI), and general control non-repressed 2 kinase (GCN2). Its phosphorylation results, among others, in the downstream inhibition of translation with accumulation of discrete foci in the cytoplasm termed stress granules (SGs). Our results indicated that USUV infection evades cellular stress response impairing eIF2α phosphorylation and SGs assembly induced by treatment with the HRI activator ArsNa. This protective effect was related with oxidative stress responses in USUV-infected cells. Overall, these results provide new insights into the complex connections between the stress response and flavivirus infection in order to maintain an adequate cellular environment for viral replication.

## Author summary

Usutu virus (USUV) infection impairs eIF2α phosphorylation and SGs assembly, in an oxidative stress related manner, as a mechanism to evade cellular stress response. Our results provide new insights into the complex connections between the stress response and USUV infection to maintain a better cellular environment for viral replication.

## Introduction

Usutu virus (USUV) is a member of the genus *Flavivirus*, within the family *Flaviviridae*, closely related to other clinically relevant flaviviruses such as West Nile virus (WNV), Japanese encephalitis virus (JEV), Zika virus (ZIKV), and dengue virus (DENV) [1]. The virus was isolated for the first time in 1959 from a female adult *Culex neavei* mosquito in South Africa [2] and since then, it has been continuously circulating in several African countries [3]. Outside Africa, USUV was first reported in Europe in 2001, where caused the massive death of blackbirds in Austria [4], although a retrospective study showed that the virus was already present in Italy in 1996 [5]. Nowadays, the circulation of USUV has been reported in 15 European countries in both animals and humans, showing an expansion in spatial distribution and host range [6]. Even more, recent evidences suggest that introduction episodes into Europe from Africa are still happening [7]. Viral transmission cycle involves mosquitoes, mainly of the *Culex* genus, and birds as amplifying hosts, which act as USUV natural reservoir and facilitate dissemination of the virus at long distances, being humans and other mammals incidental hosts [8]. There is growing evidence on the zoonotic potential of USUV as the cause of a variety of symptoms that include fever, rash and neurological diseases [3]. In fact, recent reports have confirmed the association of USUV with human cases of encephalitis and meningoencephalitis [9–17], and more recently with facial paralysis [18,19]. In addition, USUV replication in human retinal pigment epithelium has also been described [20]. There is neither vaccine nor antiviral drug to prevent or combat USUV virus licensed for human or veterinary usage, so the knowledge of the biology of this pathogen is crucial for the development of preventive and therapeutic strategies.

The replication and assembly of USUV take place on endoplasmic reticulum (ER)-derived structures [21]. Viral proteins are directly involved in membrane rearrangements of the ER required for the development of the replication complexes [22]. Flavivirus replication results in the induction of cellular stress [21,23,24], and in the specific the case of USUV, we have previously described the activation of stress-related pathways such as the unfolded protein response (UPR) [25]. However, there are still many pieces lacking to complete the puzzle of how flaviviruses interact with the integrated stress response (ISR) (reviewed in [26]). The ISR can be activated in response to several stress conditions, as amino acid starvation, viral infections, reactive oxygen species (ROS), or ER stress [27]. The core event in this pathway is the phosphorylation of eukaryotic translation initiation factor 2 alpha (eIF2α) by one of the four members of the eIF2α kinase family: protein kinase R (PKR), PKR-like endoplasmic reticulum kinase (PERK), heme-regulated inhibitor kinase (HRI), and general control non-repressed 2 kinase (GCN2). Among them, PKR is activated by viral double-stranded RNA upon replication of many RNA viruses; accumulation of unfolded proteins by the ER activates PERK; HRI is usually triggered by increased levels of ROS; and GCN2 activation is produced in response to starvation for amino acids [28,29]. Small changes in eIF2α phosphorylation can derive in a high suppression of downstream protein synthesis, and even disable the cellular translation machinery [30]. Viruses have acquired a vast number of strategies to protect eIF2α from phosphorylation by the stress-activated eIF2α kinases, allowing their replication [31]. This phosphorylation results in the downstream inhibition of translation and protein synthesis by reducing the availability of the eIF2-GTP-tRNA_i_^Met^ ternary complex [32], giving rise to the assembly of cytosolic aggregates of stalled translation preinitiation complexes called stress granules (SGs) [33,34]. Induction of SGs upon virus infection is commonly associated to activation of PKR by recognition of viral dsRNA [35]. In this regard, certain viruses, including some flaviviruses, may modulate SGs formation, either antagonizing its induction, or taking advantage of them for their own replication [36,37].

Additionally, viral infections can also trigger oxidative stress, which is induced when the balance between reactive oxygen species (ROS) production and degradation is broken in the cell [38]. ROS can act as cellular messengers in redox signaling, and oxidative stress can affect the mechanisms of cellular signaling cascades [39]. Oxidative stress induced by ROS has been confirmed as a mediator of apoptosis in virus-infected cells, and flavivirus infection may induce increased levels of ROS associated with oxidative stress [40–42]. In this study, we have analyzed the relationship between USUV infection, and the activation of three main eIF2α stress kinases (HRI, PERK and PKR), the assembly of SGs in infected cells and the role of oxidative stress in these processes.

## Methods

### Cells, viruses, and viral titrations

Vero CCL81 (ATCC CCL-81) cells were grown in Eagle’s Minimum Essential Medium (EMEM) (Lonza, Verviers, Belgium) with 5% fetal bovine serum (FBS) (Gibco, LifeTechnologies, Carlsbad, CA, US) at 37°C and 5% CO_2_. Neuro2a mouse neuroblastoma cells were grown in Dulbbecco´s Modified Minimum Essential Medium (DMEM) (Lonza) with 10% FBS. In both cases culture, medium was supplemented with 100 U/ml penicillin-streptomycin and 2 mM L-glutamine (Lonza). A derivative of the reference South African USUV strain SAAR 1776 was used [43] (Genbank KU760915.1). The multiplicity of infection (MOI) defined as plaque forming units (PFU) per cell is indicated in the figure legends. For infections in liquid medium, viral inoculum was adsorbed (1 h) to cell monolayers at 4°C to allow virus attachment, to synchronize viral entry and to avoid the effects resulting from viral spread. Then, the plates were incubated 1 h at 37°C, and viral inoculum was removed before fresh medium containing 2% FBS was added. Viral titers were determined at 24 and 48 h post-infection (p.i.) by standard plaque assay in semisolid agarose medium. To this end, 10-fold serial dilutions of the supernatants of infected cultures were added to subconfluent Vero cell monolayers grown on six-well tissue culture dishes. Viral inoculum was removed after 1 h of incubation at 37°C, and fresh medium containing 2% FBS and 1% agarose (CondaLab, Madrid, Spain) was added. Plates were incubated 72 h at 37°C and then fixed with 4% formaldehyde. Lysis plaques were counted after staining with crystal violet.

### Antibodies

Mouse monoclonal antibody J2 against double-stranded RNA (dsRNA), rabbit polyclonal serum against G3BP1, rabbit polyclonal anti-phospho-eIF2α (Ser 51), rabbit polyclonal against HRI, and mouse monoclonal anti-β-actin antibodies were purchased from Scicons (Budapest, Hungary), Genetex (Irvine, CA, USA), Cell Signaling (Danvers, MA, USA), Abcam (Cambridge, UK), and Sigma (San Luis, MO, USA), respectively. Secondary antibodies against mouse or rabbit IgGs coupled to Alexa Fluor 488, 594 or 647 were purchased from Life Technologies (Molecular Probes, Eugene, O). Anti-rabbit and anti-mouse secondary antibodies coupled to horseradish peroxidase were from Dako (Santa Clara, Ca, USA) and Sigma, respectively.

### Drug treatments

Sodium arsenite (ArsNa) (https://pubchem.ncbi.nlm.nih.gov/compound/443495), BTdCPU (https://pubchem.ncbi.nlm.nih.gov/compound/49787174), dithiothreitol (DTT) (https://pubchem.ncbi.nlm.nih.gov/compound/446094), and BEPP monohydrochloride (https://pubchem.ncbi.nlm.nih.gov/compound/2858173) were purchased from Sigma and MedChemExpress (Monmouth Junction, NJ, US), and used at 0.5 mM, 20μM, 1 mM and 10 μM, respectively. Stock solutions were prepared in dimethyl sulfoxide (DMSO), which was also used as control in non-treated cells (drug vehicle). Cells were infected, or mock-infected, and drugs, or drug vehicle, were added to the medium at 24 or 48 h p.i., kept for 4 h, removed and, then, fresh medium was added. Short time exposition of cells to the drugs to promote stress induction has been previously described [28,44–46]. The viability of cells, with or without treatment, was determined by measuring the cellular ATP content with CellTiter-Glo Luminescent Cell Viability Assay (Promega, Madison, WI, USA), according to the manufacturer protocol.

### ROS measurement

CellROX Green reagent (Molecular Probes) was used to detect oxidative stress. To this end, cells grown on glass coverslips were treated with the drugs or vehicle at 48 h p.i. Then, CellROX green Reagent was added at a final concentration of 5 μM and incubated for 30 minutes at 37°C. Medium was removed and cells were washed 3 times with phosphate buffered saline (PBS). Cells were fixed with 4% paraformaldehyde in PBS for 15 min at room temperature (RT) and washed with PBS. Nuclei were stained with To-Pro-3 (Molecular Probes) for 5 min at RT and washed 3 times with PBS. Samples were mounted with ProLong Gold antifade reagent (Thermo Fisher) and observed using a Leica TCS SPE confocal laser-scanning microscope. Images were acquired using Leica Application Suite X software. Fluorescence quantification was performed using ImageJ (http://rsbweb.nih.gov/ij/) by determination of the integrated density. More than 30 cells were counted in each of the three independent experiments performed.

### Antioxidant assay

The antioxidant activity of USUV was measured by using the Cellular Antioxidant Assay Kit (Abcam) according to the manufacturer´s protocol. Briefly, mock-infected or USUV-infected cells (MOI of 10 PFU/cell) were seeded in 96-well black fluorescence cell culture plates. At 24 h p.i. cell medium was removed and plate was washed three times with PBS. Then, cells were incubated with a cell-permeable dichloro-dihydro-fluorescein diacetate (DCFH-DA) fluorescence probe for 1 h at 37°C. Solution was removed after incubation and cells were washed again three times with PBS. Next, the free radical initiator 2,2′-Azobis(2-methylpropionamidine) dihydrochloride (AAPH) was added to create free radicals that changed DCFH-DA into dichlorofluorescein (DCF). DCF is highly fluorescent, and this fluorescence was measured every 5 minutes for an hour in a microplate reader equipped with a 480 nm excitation filter and 530 nm emission filter. Antioxidant capacity was inversely proportional to fluorescence intensity. Quercetin (25 μM) was used as control of antioxidant activity, since it inhibits the formation of free radicals and DCF in a concentration dependent manner.

### Immunofluorescence

Cells grown on glass coverslips and treated with the drugs or vehicle at different time p.i. were washed with PBS and fixed with 4% paraformaldehyde in PBS for 15 min at RT, temperature at which all further steps were conducted. Fixed cells were then washed with PBS and permeabilized (1% bovine serum albumin [BSA], 0.1% TritonX-100, 1M glycine in PBS) for 15 min. Cells were incubated with primary antibody diluted in 1% BSA in PBS for 1 hour. After washing, cells were incubated with fluorescently conjugated secondary antibody for 45 min. Nuclei were then stained with To-Pro-3 stain for 5 min and washed 3 times with PBS. Samples were mounted with ProLong Gold antifade reagent and observed using a confocal laser-scanning microscope as described above. Fluorescence quantification was performed using ImageJ (http://rsbweb.nih.gov/ij/) by determination of the integrated density for the detection of phosphorylated eIF2α (p-eIF2α), and percentage of positive cells was calculated in the case of stress granules and cell rox determinations. More than 100 cells were counted in each of the three independent experiments performed.

### Western blot

Infected, or mock-infected cells, treated or not with ArsNa, were lysed on ice in RIPA buffer (150 mM NaCl, 5 mM β-mercaptoethanol, 1% NP-40, 0.1% sodium dodecyl sulfate [SDS], 50 mM Tris-HCl pH 8) supplemented with cOmplete protease inhibitor cocktail tablets (Roche, Indianapolis, IN), phosphatase inhibitor (Thermo Fisher), and Benzonase Nuclease (Novagen, EMD Chemicals, San Diego, CA). Protein concentration was determined by Bradford assay. Equal amounts of proteins were mixed with Laemmli sample buffer, subjected to SDS-PAGE and electrotransferred onto a Polyvinylidene difluoride membrane. Then, membrane was blocked with 5% BSA in TBS 0.05% Tween-20 (TBS-Tw), incubated overnight at 4°C with primary antibodies, washed three times with TBS-Tw, and subsequently incubated with secondary antibodies coupled to horseradish peroxidase (45 min at RT) diluted in 1% BSA in TBS-Tw. Next, membrane was washed three times and proteins were detected by chemiluminescence using a ChemiDoc XRS+ System (Bio-Rad, Hercules, CA). Intensity of protein bands was quantified using ImageLab software 2.0.1 (Bio-Rad).

### Flow cytometry

For two-colour staining, mock-infected, or USUV-infected cells (MOIs of 1 and 10 PFU/cell) treated with DMSO at 24 h p.i. were harvested using trypsin, washed with PBS, and fixed with 4% paraformaldehyde in PBS for 15 min at RT. Fixed cells were washed with PBS and permeabilized (1% bovine serum albumin [BSA], 0.1% TritonX-100, 1M glycine in PBS) for 15 min at RT. Cells were then incubated with primary antibodies diluted in FACS buffer (0.1% BSA, 0.01% sodium azide in PBS) for 30 min at RT. After washing in FACS buffer, cells were incubated with fluorescently conjugated secondary antibody for 30 min at RT. Subsequently, cells were washed prior to analysis by flow cytometry, using a FACSCanto II cytometer (Becton Dickinson, San Jose, CA). Sample analysis was performed with FlowJo 10.7.1

### Statistical analyses

Data were presented as mean ± standard deviation of the mean (SD). To test the significance of the differences, analysis of the variance (ANOVA) was performed with GraphPad Prism 6 for Windows (GraphPad Software Inc., San Diego, CA) with Bonferroni’s correction for multiple comparisons. Statistically significant differences were considered when *P*<0.05.

## Results

### USUV infection inhibits ArsNa-induced eIF2α phosphorylation

**Fig 1:**
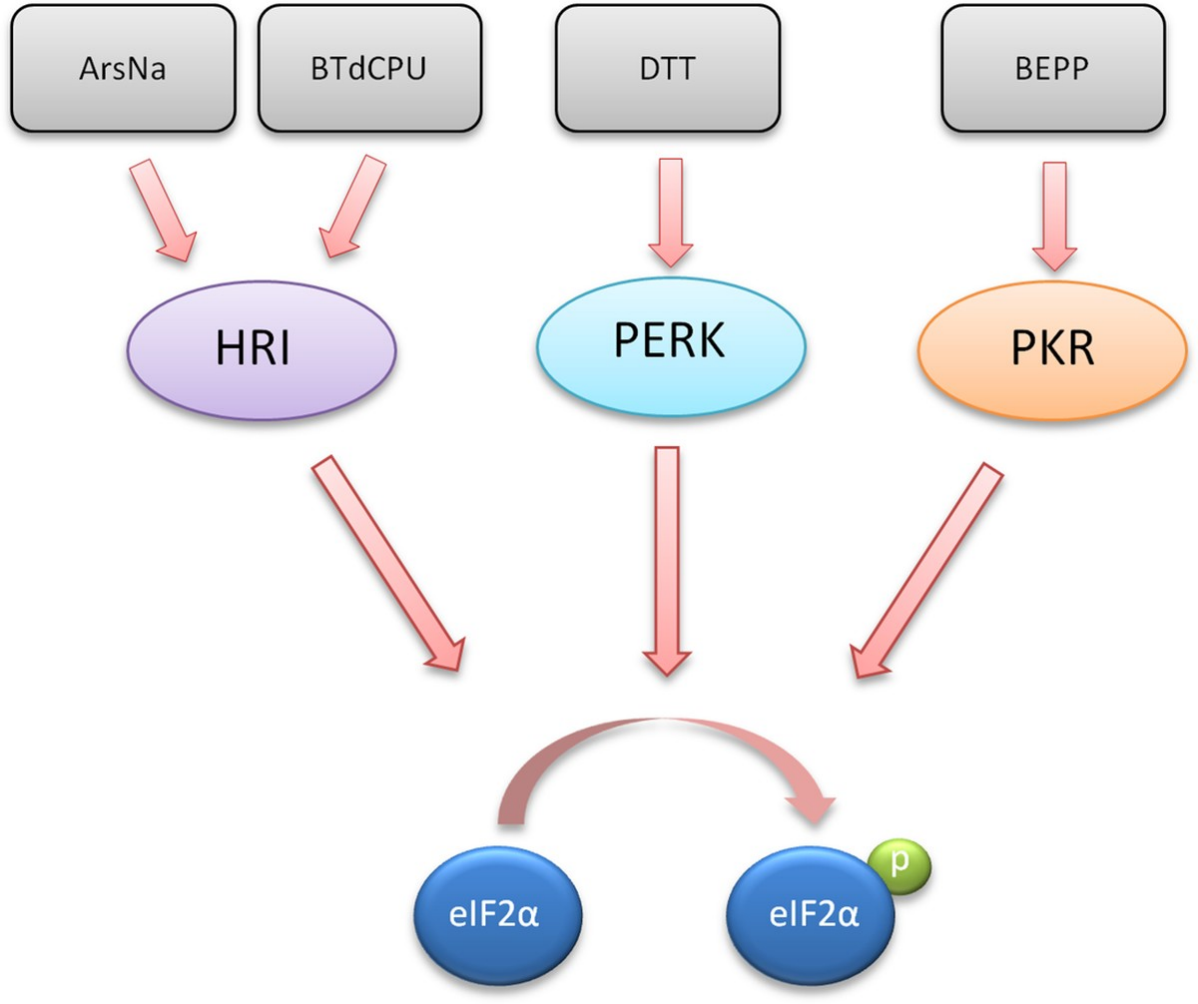
Stress kinase activators and phosphorylation of eIF2α. The chemical inducers used in this study (ArsNa, BTdCPU, DTT, and BEPP) and its target cellular kinase (HRI, PERK, and PKR) leading to eIF2α phosphorylation are indicated.

**Fig 2:**
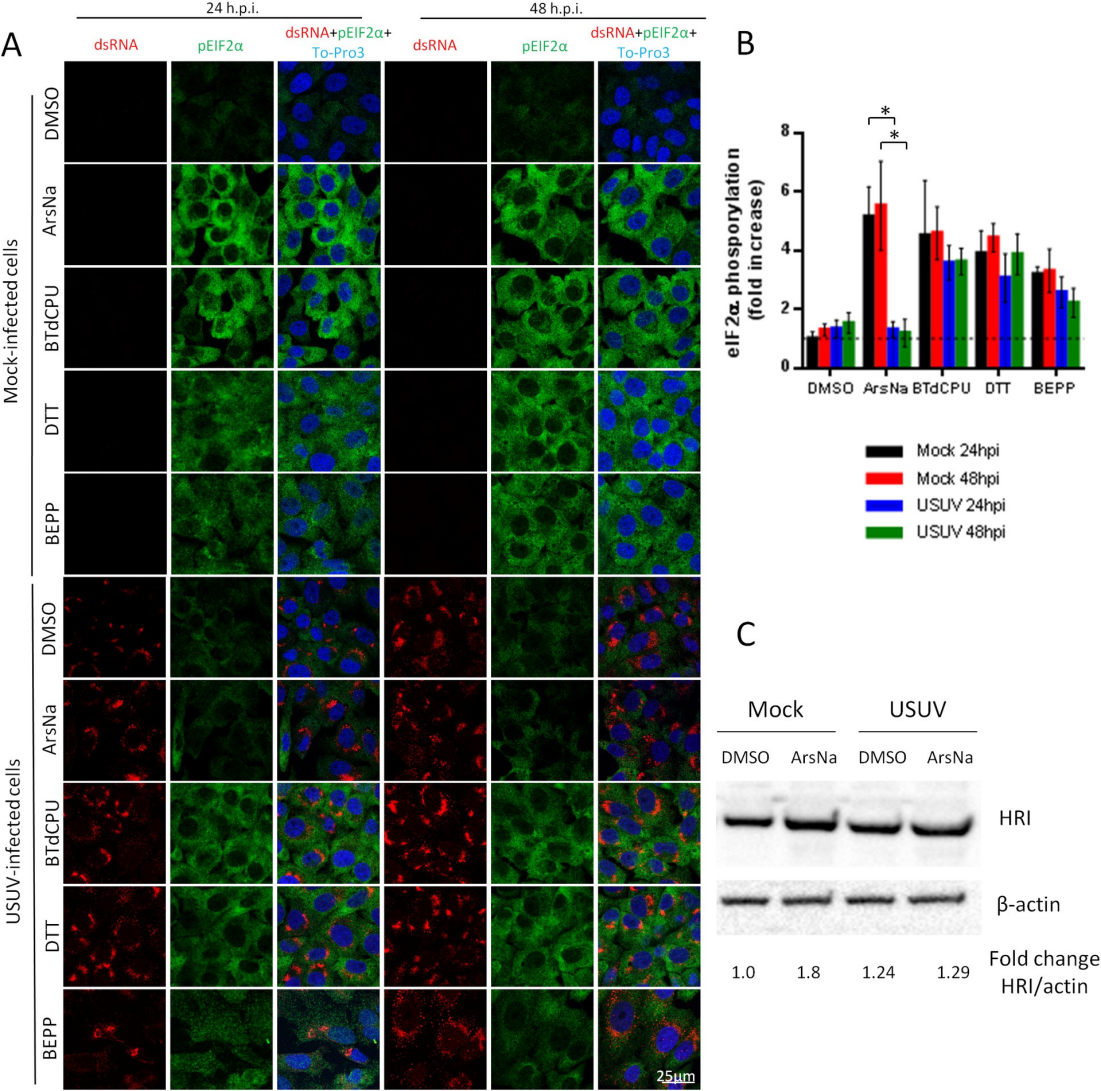
USUV infection inhibits ArsNa-induced eIF2α phosphorylation in Vero cells. (A) Vero cells were mock or USUV infected (MOI of 1), treated with ArsNa (0.5 mM), BTdCPU (20 μM), DTT (1 mM), or BEPP (10 μM) for 4 hours at 24 and 48 h p.i., and analyzed by immunofluorescence. Phosphorylated eIF2α was detected with anti-p-eIF2α antibody (green) and USUV-infected cells with anti-dsRNA antibody (red). Nuclei were stained with To-Pro-3 (blue). (B) Quantification of p-eIF2α fluorescence intensity in cells treated as in (A). Scale bars, 25 μm. Statistically significant differences were considered when *P*<0.05 and marked with an asterisk.(C) Vero cells were mock or USUV infected at an MOI of 1, and treated with ArsNa (0.5 mM) or the with same amount of DMSO for 4 hours at 48 h p.i. Cells were lysed and HRI was detected by western blot using an specific antibody. Membrane was reincubated with an anti-β-actin antibody as a control for protein loading.

**Fig 3:**
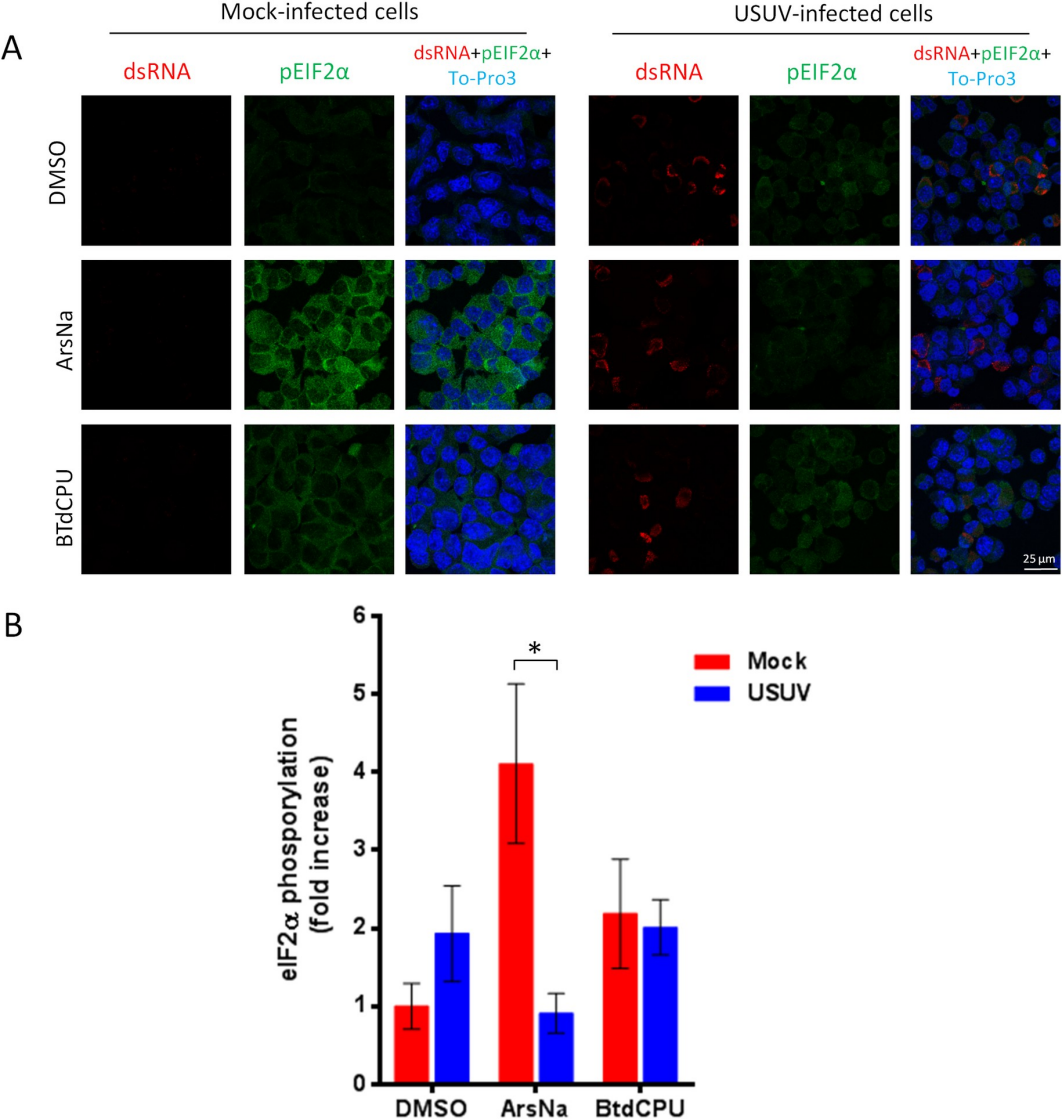
USUV infection inhibits ArsNa-induced eIF2α phosphorylation in Neuro2a cells. (A) Neuro2a cells were mock or USUV infected (MOI of 1), treated with ArsNa (0.5 mM) or BTdCPU (20 μM), for 4 hours at 48 h p.i., and analyzed by immunofluorescence. Phosphorylated eIF2α was detected with anti-p-eIF2α antibody (green) and USUV-infected cells with anti-dsRNA antibody (red). Nuclei were stained with To-Pro-3 (blue). (B) Quantification of p-eIF2α fluorescence intensity in cells treated as in A. Scale bars, 25 μm. Statistically significant differences were considered when *P*<0.05 and marked with an asterisk.

Cells may respond to different stressors, including viral infections, by inducing phosphorylation of eIF2α. However, there is a controversy on the induction of eIF2α phosphorylation upon flavivirus infection with evidences for [47] and against it [34]. Therefore, we analyzed the phosphorylation of eIF2α in USUV-infected Vero cells, the most commonly used model in USUV investigations [48], by immunofluorescence using specific antibodies against phosphorylated eIF2α (p-eIF2α) at serine 51. As most African and European USUV isolates have a similar pathogenicity in mammalian models [49,50], infections were performed with a virus isolate with proved pathogenicity in these models [43,51]. To evaluate whether USUV was affecting the signalling of stress kinases in charge of eIF2α phosphorylation, its phosphorylation was induced using pharmacological activators of stress kinases (Fig 1). Mock-infected or USUV-infected Vero cells were treated with ArsNa or BTdCPU, which activate the HRI [52,53], DTT to activate PERK [54], or BEPP to activate PKR [55] kinases to phosphorylate eIF2α, or with the same amount of drug vehicle (DMSO). Following 4 h of exposure to the treatments, detection of p-eIF2α in mock-infected and USUV-infected cells was performed by immunofluorescence. Infection with USUV of vehicle-treated cells did not significantly increase the levels of p-eIF2α either at 24 or at 48 h after infection when compared to mock-infected cells (Fig 2A and 2B). On the contrary, an increase in eIF2α phosphorylation was observed in mock-infected cells treated with each of the four drugs (Fig 2A and 2B). Remarkably, a high decrease in the intensity of p-eIF2α was noticed in USUV-infected cells that had been treated with ArsNa when compared to ArsNa-treated mock-infected, whereas only a non significant reduction was detected in USUV-infected cells after treatment with either BTdCPU, DTT, or with BEPP (Fig 2A and 2B). These results indicated that USUV infection prevented the phosphorylation of eIF2α through the HRI pathway activated by ArsNa. In this sense, to confirm if HRI pathway was affected after USUV infection, mock- and USUV-infected cells were treated with ArsNa or drug vehicle (DMSO), and detection of HRI kinase was carried out by western blot. An increase in the amount of HRI was only observed in mock-infected cells after treatment with ArsNa, while levels of HRI upon USUV infection were similar in cells treated or not with the drug. These results showed that infection with USUV did not enhance HRI expression. Furthermore, USUV ability to counteract the raise of HRI levels after treatment with ArsNa was observed (Fig 2C). Considering that no significant eIF2α phosphorylation was observed upon USUV infection, different MOIs of the virus were tested by immunofluorescence and cell flow cytometry (S1 Fig). No differences were also observed at any of the MOIs tested by either immunofluorescence (S1A and S1B Fig) or by flow cytometry (S1C and S1D Fig), thus confirming our previous results. Moreover, to evaluate whether the phosphorylation of eIF2α was dependent on the cell line, ArsNa and BTdCPU were tested in Neuro2a cells, a neural origin cell line (Fig 3). The decrease in the intensity of p-eIF2α in USUV-infected cells treated with ArsNa when compared to ArsNa-treated mock-infected, and the no significant reduction after treatment with BTdCPU were similar to that observed in Vero cells (Fig 3A and 3B). Considering that ArsNa and BTdCPU phosphorylate eIF2α by activating HRI, the differences observed between both drugs may seem somewhat surprising. However, differences in the induction of oxidative stress in cells treated with these two compounds have been previously reported, since BTdCPU provides eIF2α phosphorylation without causing oxidative stress [52], whereas ArsNa is considered an oxidative stress inducer [56]. Overall, these results suggest that USUV infection mainly prevented the phosphorylation of eIF2α by the kinase HRI related to oxidative stress, whereas other pathways leading to eIF2α phosphorylation, such as PERK or PKR, were less affected upon infection with this virus.

### ROS and USUV infection

**Fig 4:**
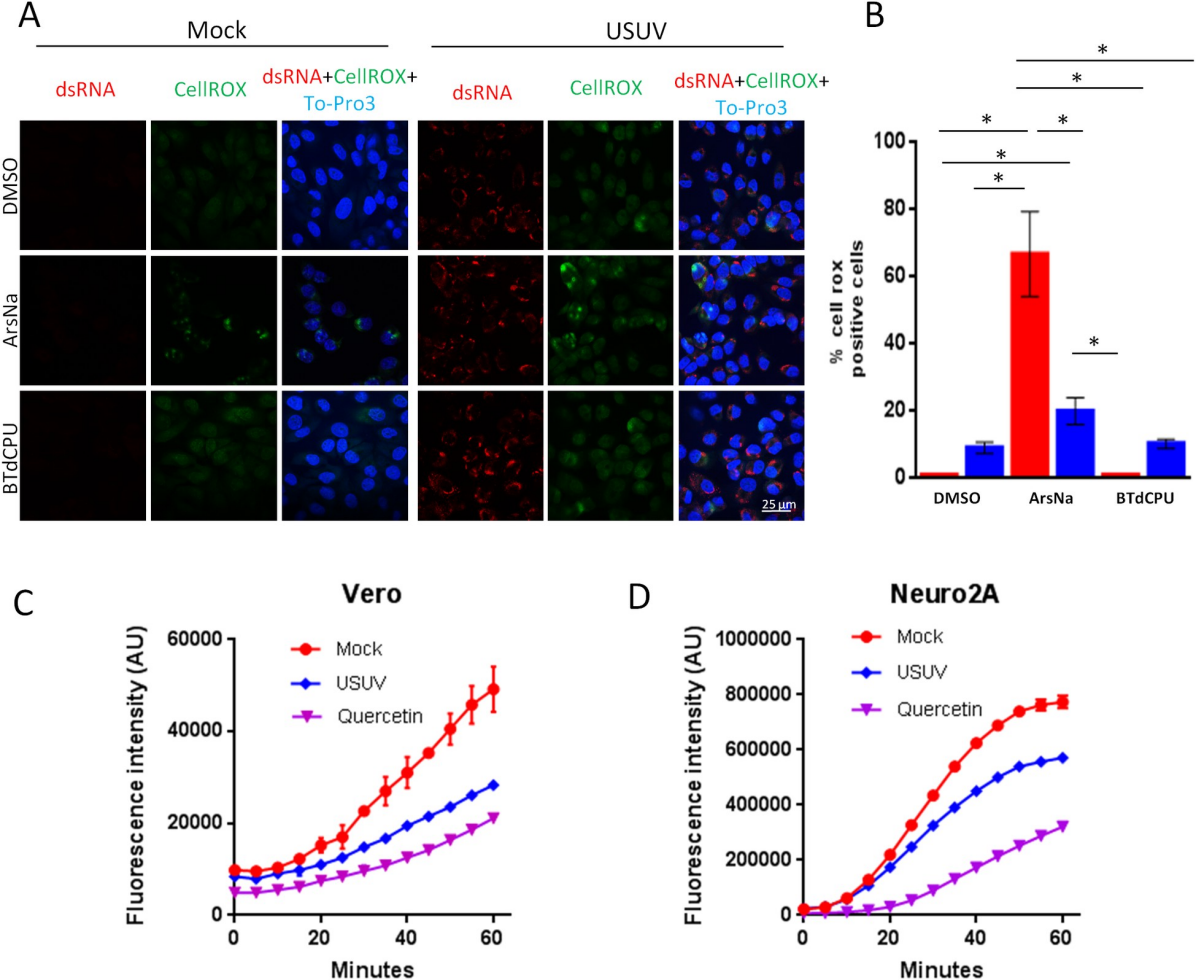
ROS in USUV infection. (A) Vero cells were mock-infected or infected with USUV (MOI of 1) and then treated with drug vehicle (DMSO), ArsNa (0.5 mM) or BTdCPU (20μM) for 4 hours at 48 h p.i. CellROX green Reagent was added at a final concentration of 5 μM and incubated for 30 minutes at 37°C, and cells were analyzed by immunofluorescence. Green fluorescent signal showed the ROS-mediated oxidation of the reagent. Nuclei were stained with To-Pro-3 (blue). Scale bars, 25 μm. (B) Quantification of CellROX fluorescent puncta in cells treated as in A. Data are means of three independent experiments. Statistically significant differences were considered when *P*<0.05 and marked with an asterisk. (C and D) Antioxidant activity of USUV infection. (C) Vero and (D) Neuro2a cells were mock-infected or infected with USUV at an MOI of 10 and left untreated. The conversion of DCFH-DA into DCF upon addition of a free radical initiator was measured by recording the fluorescence in a plate reader every 5 min. Antioxidant capacity was inversely proportional to fluorescence intensity. Quercetin (25 μM) was used as control of antioxidant activity. See methods for details.

Oxidative stress is induced when generation and degradation of ROS are unbalanced [26,38], and eIF2α kinases can be activated in response to ROS production. Flavivirus infection has been previously reported to induce ROS production [40–42], although ZIKV, WNV, DENV, and JEV infected cells were reported to develop resistance to ArsNa induced oxidative stress [28,33,57]. To analyze the generation of ROS in response to HRI activators upon USUV infection, cells were mock or USUV infected and treated with either drug vehicle (DMSO), ArsNa, BTdCPU, or left untreated. Cells were then incubated with CellROX Green Reagent to detect ROS generation and observed by immunofluorescence. An increase in ROS staining was detected in USUV infected cells in comparison to uninfected cells treated with drug vehicle, confirming that USUV infection induced oxidative stress (Fig 4 and 4B). This increase was similar in vehicle-treated and BTdCPU-treated cells infected with USUV virus, and no differences were appreciated between vehicle-treated and untreated cells (S2 Fig). However, in the case of ArsNa, USUV infection significantly reduced the production of ROS, thus evidencing an antioxidant protective effect of USUV against external induction of oxidative stress induced by ArsNa. Hence, differences in oxidative stress effect could explain the observed differences in the inhibition of eIF2α phosphorylation after USUV infected cells treatment with the two different HRI activators. These results could somehow indicate that although USUV infection results in some oxidative stress, infected cells have the ability to counteract that induced by ArsNa. In fact, the increase in ROS levels induced by WNV was counterbalanced by a potent virus-induced antioxidant response sufficient to overcome that induced by ArsNa treatment [28]. Accordingly, a similar behavior could explain the differences observed between uninfected and USUV-infected ArsNa-treated cells. To assess it, an antioxidant assay to explore the potential antioxidant response in USUV-infected cells against external oxidative agents was performed. Thereby, USUV-infected untreated cells were incubated with the fluorimetric probe DCFH-DA [58], and, next, a free radical initiator was added to create free radicals that changed DCFH-DA into dichlorofluorescein (DCF), which is highly fluorescent, and the fluorescence intensity was recorded over time (Fig 4C and 4D). This antioxidant effect was inversely proportional to fluorescence increase. In this assays, the potent antioxidant quercetin was used as control of antioxidant activity [59,60]. Results showed that USUV exhibited an antioxidant capacity in both Vero (Fig 4C) or Neuro2a (Fig 4D) cell lines. Taken together, these results demonstrate the antioxidant capability of USUV to counteract the activation of oxidative stress produced by external agents such as ArsNa.

### USUV infection inhibits ArsNa-induced SGs formation

**Fig 5:**
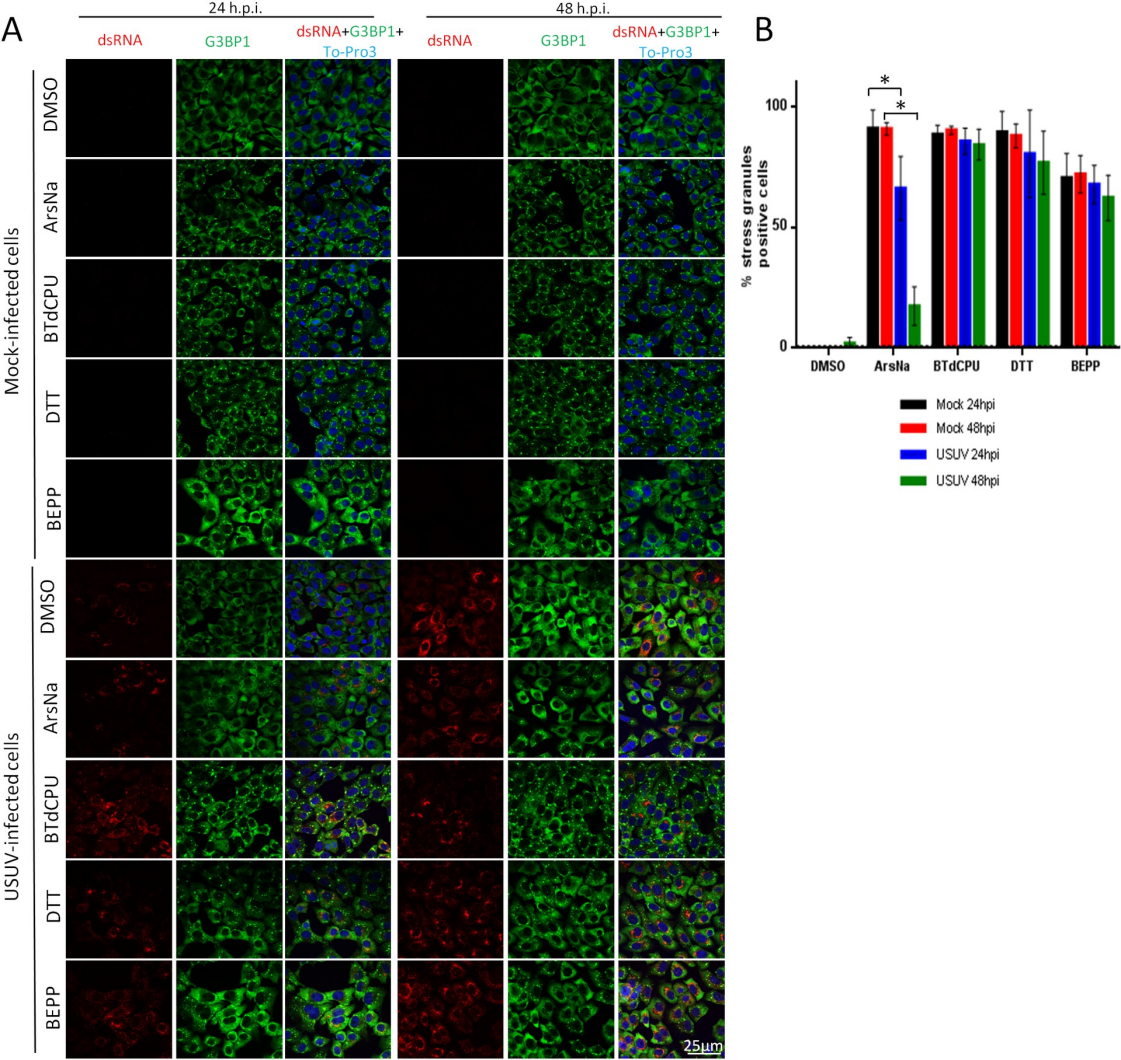
USUV infection inhibits ArsNa-induced SGs formation. (A) Vero cells were mock- or USUV- infected at an MOI of 1, treated with ArsNa (0.5 mM), BTdCPU (20μM), DTT (1mM) or BEPP (10 μM) for 4 hours at 24 and 48 h p.i., and analyzed by immunofluorescence. SGs were detected with anti-G3BP1 antibody (green) and USUV-infected cells with anti-dsRNA antibody (red). Nuclei were stained with To-Pro-3 (blue). (B) Quantification of SG-positive cells in cells treated as in A. Scale bars, 25 μm. Statistically significant differences were considered when *P*<0.05 and marked with an asterisk.

The activation of the cellular stress response results in the accumulation of large cytoplasmic foci nucleated by the aggregation of untranslated messenger ribonucleoproteins (mRNPs) termed stress granules (SGs). These structures are mainly composed of non-translating mRNAs, stalled translation initiation complexes, and RNA-binding proteins. SGs participate in post-transcriptional regulation of gene expression by controlling RNA translation and stability [61,62]. The formation of SG can be mediated by phosphorylation of eIF2α. However, certain viruses, including many flaviviruses, have the ability to antagonize SG formation during infection, while others manipulate SG responses for their own benefit during replication [33,34,57,63–65]. As SGs contain RNA-binding proteins, such as PABP, TIA 1, TIAR, and G3BP1 [66], the presence of the SG-nucleating RNA-binding G3BP1 protein was assayed by immunofluorescence in mock-infected and USUV-infected cells. Cells were treated with stress kinase activators (ArsNa, BTdCPU, DTT, or BEPP) to promote the formation of SGs, or with the same amount of drug vehicle (DMSO). As expected, treatment with stress kinase activators resulted in an increase in the proportion of cells showing relocation of G3BP1 to SGs in their cytoplasm. Accordingly, aggregates of G3BP1 protein (SGs) were observed in the cytoplasm in more than 90% of mock-infected cells treated with ArsNa, BTdCPU, or DTT, and around 70% in those treated with BEPP, while no SGs were detected in vehicle treated cells (Fig 5A and 5B). USUV infection did not induce SG formation at 24 h p.i., and only a slight number of G3BP1 foci were detected at 48 h p.i. Remarkably, USUV-infection induced a significant reduction in the number of cells with ArsNa-induced SGs at 24 h p.i., showing even a sharper decrease in the number of cells containing ArsNa-induced SGs at 48 h p.i. (Fig 5A and 5B). However, in a similar manner to that observed for eIF2α phosphorylation (Fig 2), USUV-infection did not prevent SGs induction by other HRI activator (BTdCPU). In those cells treated with the PERK activator DTT, only a slight non-statistically significant reduction in the percentage of positive SG cells was detected at either early (24 h p.i.) or late time (48 h p.i.) after infection. In the case of SGs assembly induced by treatment with the PKR activator BEPP, no significant changes were observed at 24 or 48 h p.i. compared to mock-infected cells (Fig 5A and 5B). In all cases, viral replication intermediates, detected by labelling with anti-dsRNA antibodies, did not colocalize with the SG-nucleating RNA-binding protein G3BP1. Taken together, these results indicate that the inhibition of ArsNa-induced SG formation in USUV-infected cells is tightly correlated with the inhibition of ArsNa-induced eIF2α phosphorylation. As noted in the case of ROS production (Fig 4), infection with USUV can induce some cellular stress, observed by the formation of a slight amount of stress granules only at long times after infection. On the other hand, the differences observed between HRI activators could be influenced by oxidative stress in HRI activation during USUV-infection.

### Effect of HRI activators on USUV multiplication

**Fig 6:**
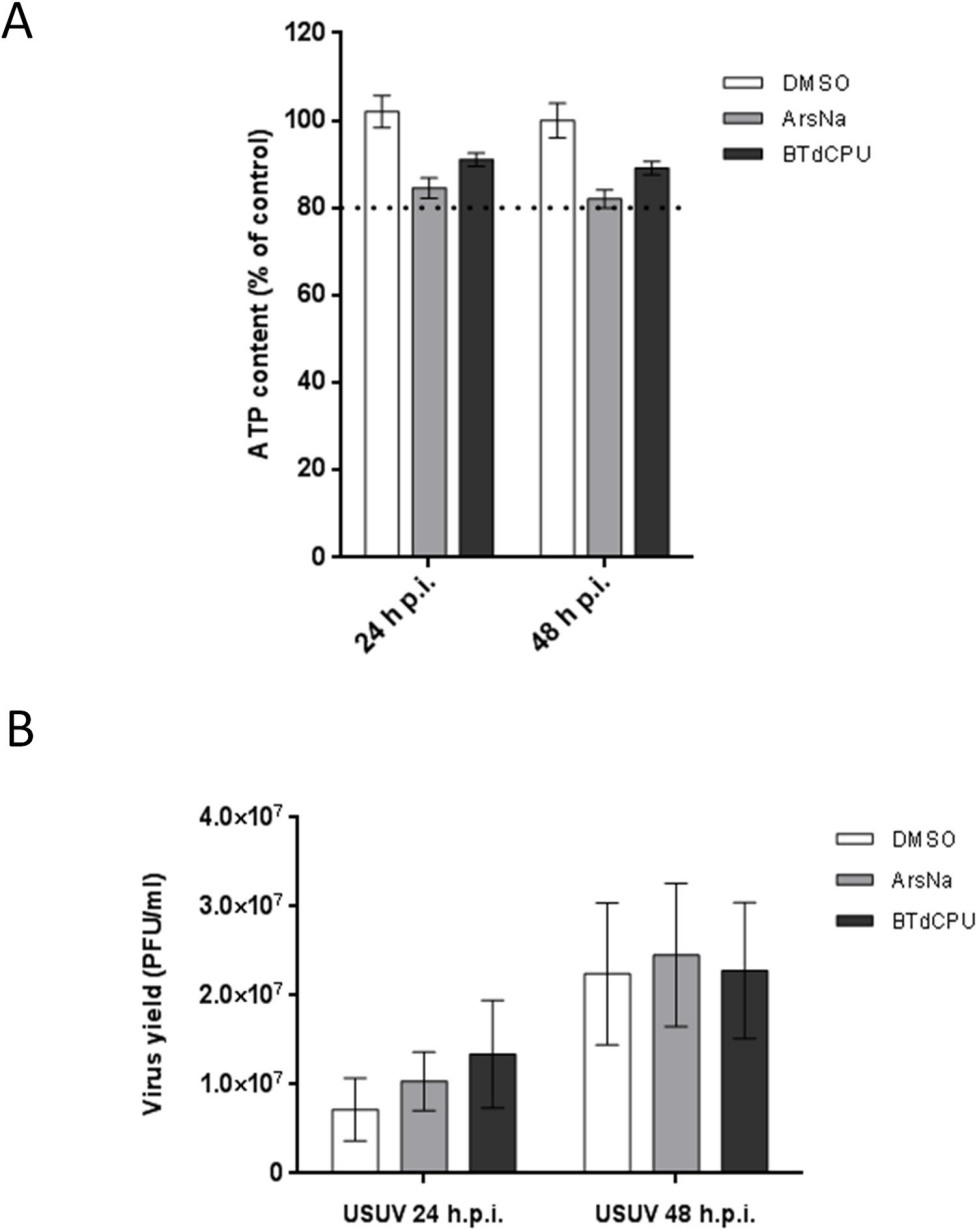
Effect of HRI activators on USUV multiplication. (A) Cell viability was determined as ATP content in mock-infected cells treated with the drugs at 24 and 48 h. The dotted line indicates 80% of the viability of control cells; (B) Cells were infected with an MOI of 1 PFU/cell, and 24 or 48 h p.i. were treated with ArsNa (0.5 mM), or BTdCPU (20 μM) for 4 hours. Then, virus yields in culture supernatants were determined by plaque assay. Data represent the average of three independent experiments.

Flaviviruses can modulate cellular stress responses for viral multiplication, including some eIF2α-specific components from SGs [21,67]. Considering that USUV-infected ArsNa treated cells showed a decrease in the levels of p-eIF2α and SGs formation (Figs 2, 3 and 5), and the observed differences in this behaviour when BTdCPU, another HRI activator, was used, the effect of these two drugs on USUV multiplication was assessed. To this end, viability of cells with or without treatment was determined. The cellular ATP content in samples treated with either 0.5 mM ArsNa or 20μM BTdCPU was higher than 80% (Fig 6A) evidencing the low cytotoxicity of the drugs at the tested concentration. Then, USUV-infected cells were treated at 24 or 48 h p.i. for 4 h with both drugs, and viral titers were determined. A non-significant increase in viral yields (Fig 6B) was observed in all treated cells at every tested time when compared to USUV infected treated with vehicle alone. Overall, these data confirmed the robustness of the virus exerted mechanisms to counteract cell stress responses in order to maintain the adequate cellular environment necessary for viral replication.

## Discussion

Little is known about the cellular pathways involved in USUV replication and pathogenesis. It has been reported that USUV replication takes place associated to the ER of infected cells [25,68], triggering intracellular responses related to cellular stress such as the unfolded protein response (UPR) and autophagy [25], activating innate immunity [69], and resulting in cell death [68,70], but many other aspects of its replication process are unknown, as the cellular stress response to USUV infection. The mechanisms in which viruses cope with cellular stress induced by infection and the ISR are important to understand how the virus can efficiently multiply and induce pathogenesis [71–75]. Accordingly, and since involvement of stress eIF2α in flavivirus infections is still controversial [34,76–78], we have explored the interaction of USUV with the ISR. For this purpose, we focus on the involvement of stress eIF2α, the key transcriptional factor that governs the ISR to different cellular stressors, such as hypoxia, amino acid or glucose deprivation, and viral infection, as well as cell intrinsic stresses, such as oxidative and ER stress and UPR [26,79,80]. Our data demonstrated that USUV infection did not provoke a sustained increase in the levels of p-eIF2α, which is consistent with previous reports regarding mosquito-borne flaviviruses, as WNV [57,76], or ZIKV [34], even though, as mentioned above, it is not a common feature among flaviviruses, since other members of this genus, such as TBEV, showed an induction of eIF2α phosphorylation upon infection [47,77]. In the case of DENV, there are some conflicting reports, with authors reporting an upregulation of eIF2α phosphorylation [78,81] and other not [34]. Additionally, there are data indicating that eIF2α phosphorylation is only transiently activated at the early stage of infection [82,83]. Moreover, our results show that USUV infection significantly inhibits eIF2α phosphorylation induced by treatment with the cellular kinase HRI inducer ArsNa, as previously reported for other flaviviruses [28,34]. However, strikingly, no significant differences were observed after treatment with another HRI inducer, BTdCPU. This could be due to the different behavior of these two drugs in the induction of oxidative stress, since BTdCPU activates eIF2α phosphorylation without causing oxidative stress [52], whereas ArsNa is considered an oxidative stress inducer [56]. Thereby, the raised levels of ROS could produce a cellular stress that somehow counteracts the phosphorylation of eIF2α after ArsNa treatment. In this regard, accumulating evidences have linked the oxidative stress to the pathogenesis of these vector-borne viruses [35], as flavivirus infections induce high levels of ROS, typically associated with oxidative stress [41,42]. However, this increase does not exhibit some characteristic effects of stress in cells, such as SG assembly or mitochondrial damage [28], thus, supporting that flavivirus infection upregulates both oxidative stress and the antioxidant response in cells [28].

No significant differences were either observed when other eIF2α phosphorylation pathways such as PERK or PKR were analyzed. In this sense, different responses related to PERK activation has been reported for flaviviruses. For instance, it has been described that the high pathogenic WNV NY-99 strain upregulated the PERK pathway [84], whilst the less pathogenic WNV Kunjin did not [85]. Conversely, while the tick-borne flavivirus Langat virus (LGTV) strongly activated this pathway, no effect on PERK upregulation was observed with the more pathogenic Powassan virus (POWV) [86]. Upregulation of this pathway has also been described for other members of the *Flaviviridae* family, such as ZIKV [87], bovine viral diarrhea virus (BVDV) [88], hepatitis C virus (HCV) [89], and DENV [90], although in the latter two activation and suppression of the PERK pathway in a time dependent manner has been reported [83]. Even more, a role of PERK, but not of PKR, in early induction and suppression of eIF2α phosphorylation upon DENV infection has been documented [83]. It should be noted that, apart from flaviviruses, other viruses use multiple strategies either to prevent [91–94], or to induce eIF2α phosphorylation [75,88,95,96] taking advantage of it for replication.

Phosphorylation of eIF2α hampers the formation of the three-subunit complex eIF2-GTP-tRNAiMet causing stalled translation initiation complexes that are assembled into SGs. Our results showed that USUV infection did not induce SGs formation at 24 h p.i., and only a slight number of G3BP1 foci were detected at 48 h p.i, possibly because of its ability to generate some ROS upon infection. However, a significant reduction in SGs formation was observed in ArsNa treated USUV-infected cells at the different time points tested when compared to mock-infected treated ones, thus confirming the close association with the inhibition of ArsNa-induced eIF2α phosphorylation. Inhibition of the formation of SGs under oxidative stress conditions following treatment with the oxidative inducer ArsNa has been reported for other flaviviruses [28,33,34,57,63], being specific to eIF2α phosphorylation in the case of ZIKV [33]. However, besides the capacity of USUV to induce an increase in levels of ROS, the virus showed a potent antioxidant activity against external stimuli.

In summary, our findings can help to improve current knowledge of USUV host cell interactions. Inhibition of SG assembly in a p-eIF2α dependent way upon USUV infection, and its relation with oxidative stress and ROS production, is provided. The antioxidant activity of the virus against external oxidative factors is also demonstrated, thus contributing to unravel the mechanisms by which the virus can counterbalance the effects of oxidative agents such as ArsNa. These data suggest that the virus can take advantage of these mechanisms to counteract cell stress responses, promoting an appropriate environment for viral multiplication. All this contribute to a better understanding of these interactions, which may be useful to elucidate the biology and pathogenesis of USUV and exploit them as an antiviral strategy.

## Supporting information

S1 Fig eIF2α phosphorylation in USUV infected Vero cells. (A) Vero cells were mock or USUV infected at different MOIs of 0.1, 1, and 10, and treated at 24 hpi with DMSO or left untreated, and analyzed by immunofluorescence. Phosphorylated eIF2α was detected with anti-p-eIF2α antibody (green) and USUV-infected cells with anti-dsRNA antibody (red). Nuclei were stained with To-Pro-3 (blue). Scale bars, 25 μm. (B) Quantification of p-eIF2α fluorescence intensity in cells treated as in (A). (C) Vero cells were mock or USUV infected at different MOIs of 1 and 10 and treated at 24 hpi with DMSO. Phosphorylated eIF2α was analyzed by flow cytometry. USUV-infected cells were selected with anti-dsRNA antibody (green) and phosphorylated eIF2α was detected with anti-p-eIF2α antibody (red). Histograms shown were obtained on gates corresponding to USUV-infected cells at MOI 1 (blue line), MOI 10 (grey line), or non-infected cells (red line). 10 000 cells were acquired. (D) Quantification of phosphorylated eIF2α fluorescence intensity in non-infected cells (red bar) or USUV infected-cells (blue and grey bar) treated as in (C).
(TIF)

S2 Fig ROS in USUV infection. (A) Vero cells were mock-infected or infected with USUV at an MOI of 1 and then treated with drug vehicle (DMSO) for 4 hours at 48 hpi, or left untreated. CellROX green Reagent was added at a final concentration of 5 μM and incubated for 30 minutes at 37°C, and cells were analyzed by immunofluorescence. Green fluorescent signal showed the ROS-mediated oxidation of the reagent. Nuclei were stained with To-Pro-3 (blue). Scale bars, 25 μm. (B) Quantification of CellROX fluorescent puncta in cells treated as in A.
(TIF)
