# Supplementary figures and images for: Relevance of oxidative stress in inhibition of eIF2 alpha phosphorylation and stress granules formation during Usutu virus infection
Source: PLoS Negl Trop Dis. 2021 Jan 25;15(1):e0009072. doi: 10.1371/journal.pntd.0009072 (PMC7861526; doi:10.1371/journal.pntd.0009072)

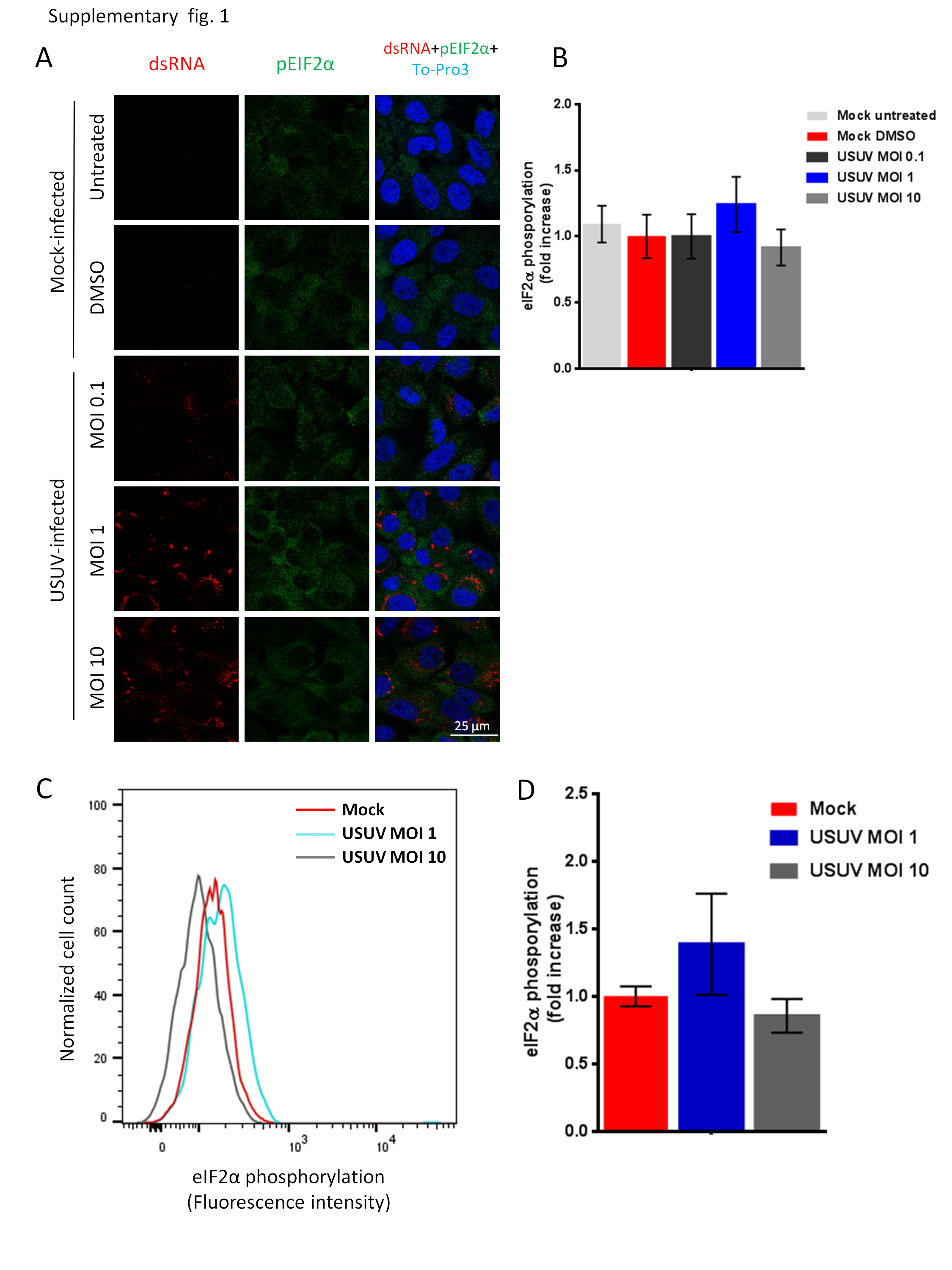

Supplement: S1 Fig — (A) Vero cells were mock or USUV infected at different MOIs of 0.1, 1, and 10, and treated at 24 hpi with DMSO or left untreated, and analyzed by immunofluorescence. Phosphorylated eIF2α was detected with anti-p-eIF2α antibody (green) and USUV-infected cells with anti-dsRNA antibody (red). Nuclei were stained with To-Pro-3 (blue). Scale bars, 25 μm. (B) Quantification of p-eIF2α fluorescence intensity in cells treated as in (A). (C) Vero cells were mock or USUV infected at different MOIs of 1 and 10 and treated at 24 hpi with DMSO. Phosphorylated eIF2α was analyzed by flow cytometry. USUV-infected cells were selected with anti-dsRNA antibody (green) and phosphorylated eIF2α was detected with anti-p-eIF2α antibody (red). Histograms shown were obtained on gates corresponding to USUV-infected cells at MOI 1 (blue line), MOI 10 (grey line), or non-infected cells (red line). 10 000 cells were acquired. (D) Quantification of phosphorylated eIF2α fluorescence intensity in non-infected cells (red bar) or USUV infected-cells (blue and grey bar) treated as in (C). (TIF) [file pntd.0009072.s001.tif]

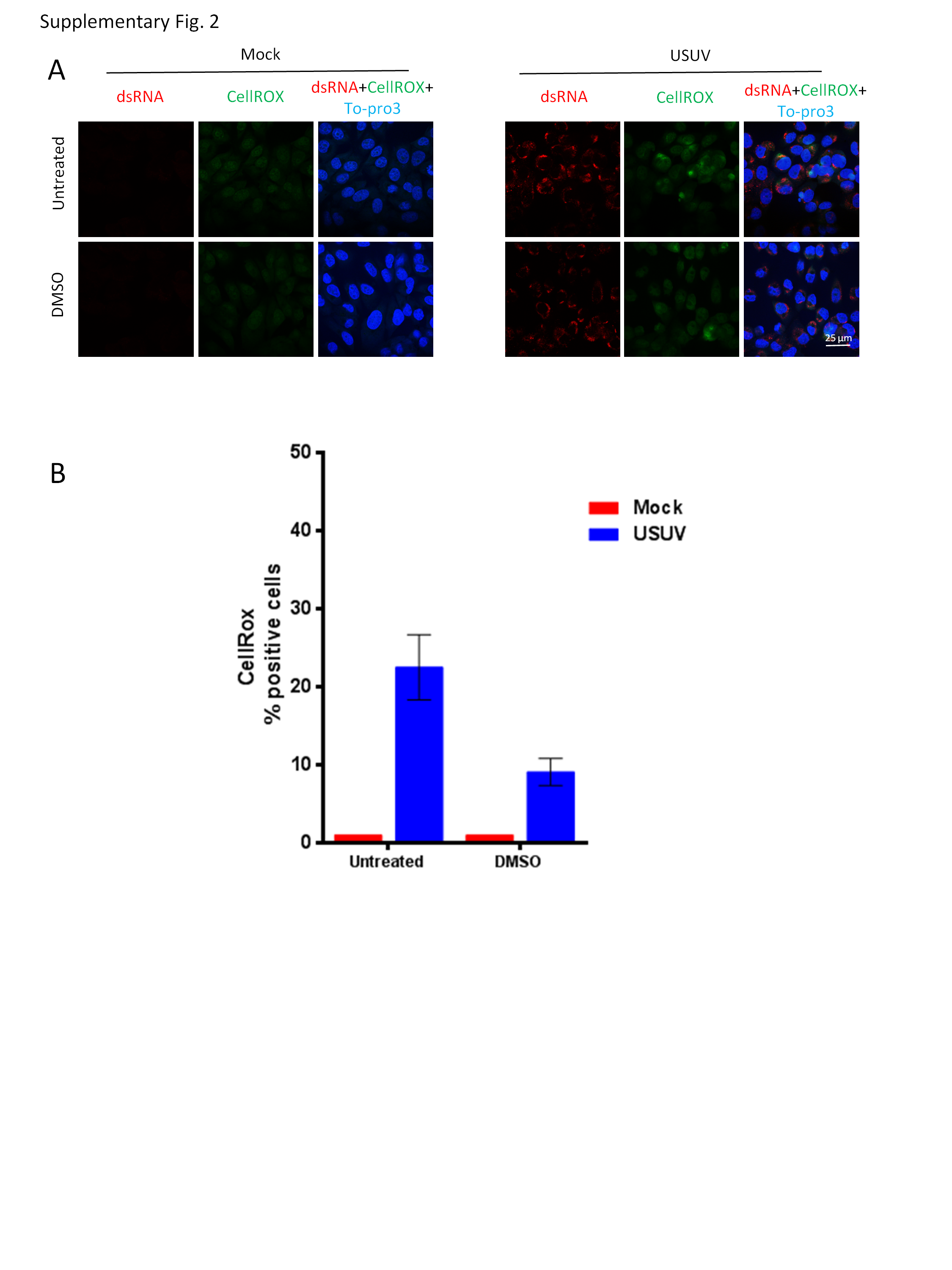

Supplement: S2 Fig — (A) Vero cells were mock-infected or infected with USUV at an MOI of 1 and then treated with drug vehicle (DMSO) for 4 hours at 48 hpi, or left untreated. CellROX green Reagent was added at a final concentration of 5 μM and incubated for 30 minutes at 37°C, and cells were analyzed by immunofluorescence. Green fluorescent signal showed the ROS-mediated oxidation of the reagent. Nuclei were stained with To-Pro-3 (blue). Scale bars, 25 μm. (B) Quantification of CellROX fluorescent puncta in cells treated as in A. (TIF) [file pntd.0009072.s002.tif]
